# Rapamycin-induced miR-21 promotes mitochondrial homeostasis and adaptation in mTORC1 activated cells

**DOI:** 10.18632/oncotarget.19947

**Published:** 2017-08-04

**Authors:** Hilaire C. Lam, Heng-Jia Liu, Christian V. Baglini, Harilaos Filippakis, Nicola Alesi, Julie Nijmeh, Heng Du, Alicia Llorente Lope, Katherine A. Cottrill, Adam Handen, John M. Asara, David J. Kwiatkowski, Issam Ben-Sahra, William M. Oldham, Stephen Y. Chan, Elizabeth P. Henske

**Affiliations:** ^1^ Department of Medicine, Pulmonary and Critical Care Medicine, Brigham and Women’s Hospital and Harvard Medical School, Boston, MA, USA; ^2^ Department of Medicine, Division of Cardiology, Center for Pulmonary Vascular Biology and Medicine, Pittsburgh Heart, Lung, Blood, and Vascular Medicine Institute, University of Pittsburgh School of Medicine and University of Pittsburgh Medical Center, Pittsburgh, PA, USA; ^3^ Department of Medicine, Division of Signal Transduction, Beth Israel Deaconess Medical Center, Harvard Medical School, Boston, MA, USA; ^4^ Department of Biochemistry and Molecular Genetics, Northwestern University, Chicago, IL, USA

**Keywords:** tuberous sclerosis complex, mTORC1, rapamycin, miR-21, mitochondria

## Abstract

mTORC1 hyperactivation drives the multi-organ hamartomatous disease tuberous sclerosis complex (TSC). Rapamycin inhibits mTORC1, inducing partial tumor responses; however, the tumors regrow following treatment cessation. We discovered that the oncogenic miRNA, miR-21, is increased in Tsc2-deficient cells and, surprisingly, further increased by rapamycin. To determine the impact of miR-21 in TSC, we inhibited miR-21 *in vitro*. miR-21 inhibition significantly repressed the tumorigenic potential of Tsc2-deficient cells and increased apoptosis sensitivity. Tsc2-deficient cells’ clonogenic and anchorage independent growth were reduced by ∼50% (*p*<0.01) and ∼75% (*p*<0.0001), respectively, and combined rapamycin treatment decreased soft agar growth by ∼90% (*p*<0.0001). miR-21 inhibition also increased sensitivity to apoptosis. Through a network biology-driven integration of RNAseq data, we discovered that miR-21 promotes mitochondrial adaptation and homeostasis in Tsc2-deficient cells. miR-21 inhibition reduced mitochondrial polarization and function in Tsc2-deficient cells, with and without co-treatment with rapamycin. Importantly, miR-21 inhibition limited Tsc2-deficient tumor growth *in vivo*, reducing tumor size by approximately 3-fold (*p*<0.0001). When combined with rapamcyin, miR-21 inhibition showed even more striking efficacy, both during treatment and after treatment cessation, with a 4-fold increase in median survival following rapamycin cessation (*p*=0.0008). We conclude that miR-21 promotes mTORC1-driven tumorigenesis *via* a mechanism that involves the mitochondria, and that miR-21 is a potential therapeutic target for TSC-associated hamartomas and other mTORC1-driven tumors, with the potential for synergistic efficacy when combined with rapalogs.

## INTRODUCTION

Tuberous sclerosis complex (TSC) is an autosomal dominant tumor suppressor gene syndrome associated with hamartomatous tumors of the brain, heart, skin and kidney, and progressive cystic lung disease lymphangioleiomyomatosis (LAM) [1, 2]. Both children and adults with TSC can also develop malignant renal cell carcinomas.

The TSC proteins, TSC1 (hamartin) and TSC2 (tuberin), function within a complex to inhibit mammalian target of rapamycin complex 1 (mTORC1) *via* the small GTPase Rheb [2-6].

In TSC patients, pivotal clinical trials have demonstrated that treatment with rapamycin or related mTORC1 inhibitors (Rapalogs) yields partial and sustained responses of kidney tumors (angiomyolipomas), brain tumors (subependymal giant cell astrocytomas, SEGA), and LAM. However, tumors regrow promptly when treatment is discontinued, and continuous, lifelong treatment appears to be necessary [7-11].

MicroRNAs (miRNA or miRs) are small RNA species that regulate gene expression by promoting target degradation or translation inhibition. Small changes in miRNA levels, even as little as 1.2-fold, can have large downstream effects, since a single miRNA can regulate dozens of genes. Selected miRNA, termed “oncomiRs,” function like oncogenes and promote cell survival, growth, proliferation, migration, and invasion in benign and malignant diseases [12].

We previously screened 946 miRNA in TSC2-deficient patient-derived angiomyolipoma cells treated for 24 hours with rapamycin or control, and identified 18 upregulated and 8 downregulated “Rapa-miRs” [13]. Surprisingly, the most strongly upregulated rapamycin-dependent miRNAs were oncomiRs, including miR-21. Prior studies have shown that miR-21 targets multiple key tumor suppressors, such as PTEN, PDCD4 and TCF21 [14-16]. miR-21 overexpression is sufficient to promote B-cell lymphoma in mice [17], the most stringent definition of an oncomiR. Expression profiling studies including The Cancer Genome Atlas (TCGA) of renal cell carcinomas have demonstrated strong correlations between higher miR-21 expression and worse prognostic outcomes [18]. Furthermore, TSC brain lesions have increased miR-21 levels by *in situ* hybridization [19]. Based on these data, we hypothesized that miR-21 may play a critical role in promoting tumorigenesis in TSC and limiting the cytotoxic effects of mTORC1 inhibition by rapalogs.

In this study, we discovered that miR-21 acts as a critical regulator of mTORC1-driven tumorigenesis, which may be supported *via* miR-21 dependent regulation of mitochondrial function and adaptation. We demonstrate that miR-21 is increased approximately 10-fold in Tsc2-deficient cells compared to wildtype controls and further induced by rapamycin. Using both *in vitro* and *in vivo* assays, we found that miR-21 supports tumorigenic growth and limits apoptosis. The effect of miR-21 inhibition in combination with rapamycin significantly reduced colony formation in soft agar. Using expression profiling coupled with a network biology-driven bioinformatic analysis, we found that miR-21 impacts mitochondrial genes in Tsc2-deficient cells. We show that miR-21 supports mitochondrial function and adaptation to rapamycin treatment. Finally, in an *in vivo* xenograft mouse model, miR-21 inhibition limited Tsc2-deficient tumor growth with substantial reductions in tumor-free survival and tumor volume. Most importantly, when combined with Rapamycin, miR-21 inhibition further reduced tumor-free survival and increased median survival following rapamycin cessation. This study provides the first evidence for miR-21 targeted therapy in TSC, with potential additional relevance to the many sporadic malignancies with mTORC1 hyperactivation.

## RESULTS

### Rapamycin induces miR-21 expression in TSC2-deficient cells *in vitro* and *in vivo*

**Figure 1:**
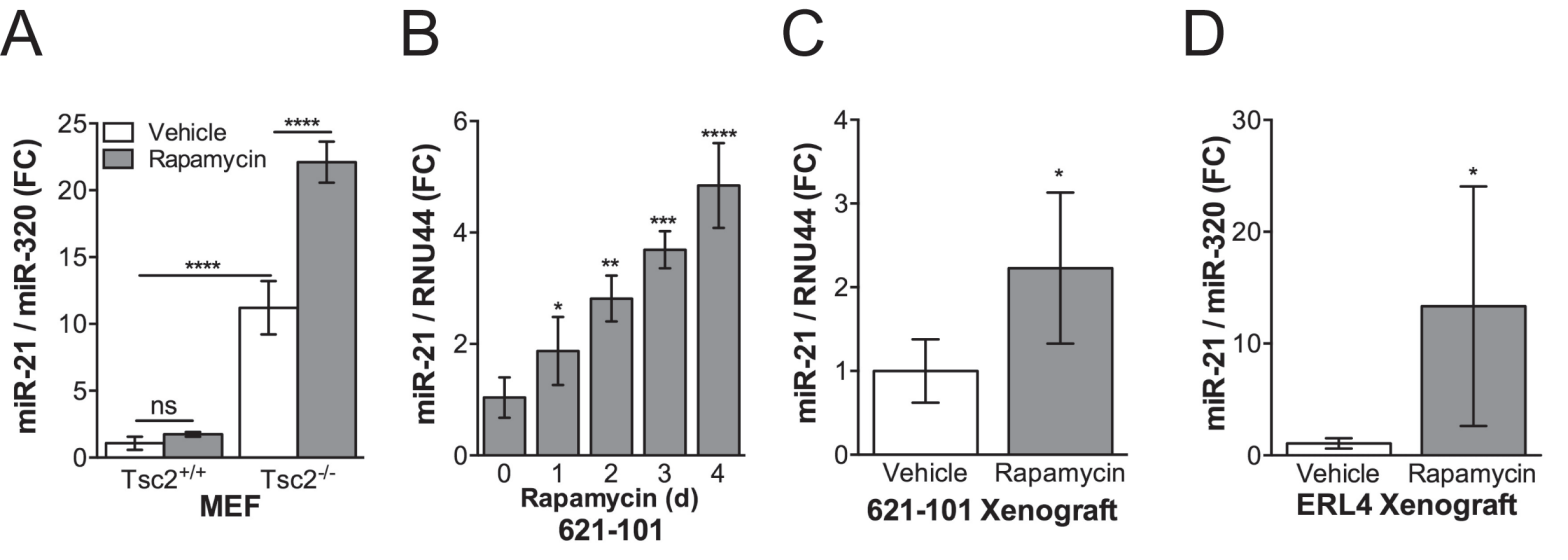
Rapamycin induces miR-21 expression in Tsc2^-/-^ cells *in vitro* and *in vivo* **A.**, miR-21 expression was assessed by qRT-PCR in Tsc2^+/+^ and Tsc2^-/-^ MEFs treated with rapamycin (20 nM, 24h) or vehicle control. **B.**, miR-21 expression was assessed in human angiomyolipoma derived 621-101 cells treated with rapamycin (20 nM) for up to 4 days. Treatment initiation was staggered so that all samples were harvested on day 4, media was changed at day 2. **C.**, Mice bearing 621-101 xenografts (*n* = 7-8) or **D.**, ERL4 xenografts (*n* = 4) that were ∼300 mm3 were treated with i.p. rapamcyin (3 mg/kg) every other day for five days. Tumors were harvested 4 hours after the final injection and miR-21 expression assessed by qRT-PCR. Data presented as mean+/- standard deviation **A.**, **B.** or 95% CI **C.**, **D.** Statistical significance was assessed by **A.**, Two-Way ANOVA with Bonferroni correction **B.**, One-Way ANOVA with Bonferroni correction or **C.** and **D.**, Mann Whitney Test with *p < 0.05, **p < 0.01, ***p < 0.001, ****p < 0.0001.

By qRT-PCR, we discovered that miR-21 expression is increased approximately 10-fold in Tsc2-deficient mouse embryonic fibroblasts (MEFs) compared to Tsc2-expressing wildtype cells (*p* < 0.0001; Figure 1A). Unexpectedly, treatment with rapamycin (20 nM, 24h) further increased miR-21 levels by approximately 2-fold in the Tsc2^*-/-*^ MEFs (*p* < 0.0001; Figure 1A). Over the course of a 4-day treatment, rapamycin induced miR-21 expression in a time dependent manner up to 5-fold (*p* < 0.0001) in TSC2-deficient 621-101 cells derived from a human patient (Figure 1B). *In vivo*, miR-21 was increased ∼2-fold (*p* < 0.05) after 1 week of rapamycin treatment (3mg/kg, MWF and harvested at 4h post final injection) in xenografts generated by subcutaneous inoculation of 621-101 cells (Figure 1C) or ERL4 (Tsc2-deficient ELT3 cells derived from an Eker rat uterine leiomyoma and stably expressing luciferase; Figure 1D) cells into immunocompromised mice. These results demonstrate that miR-21 is higher in Tsc2-deficient cells and further induced by rapamycin treatment both *in vitro* and *in vivo*.

### miR-21 promotes proliferation and resistance to apoptosis in TSC2-deficient cells

**Figure 2:**
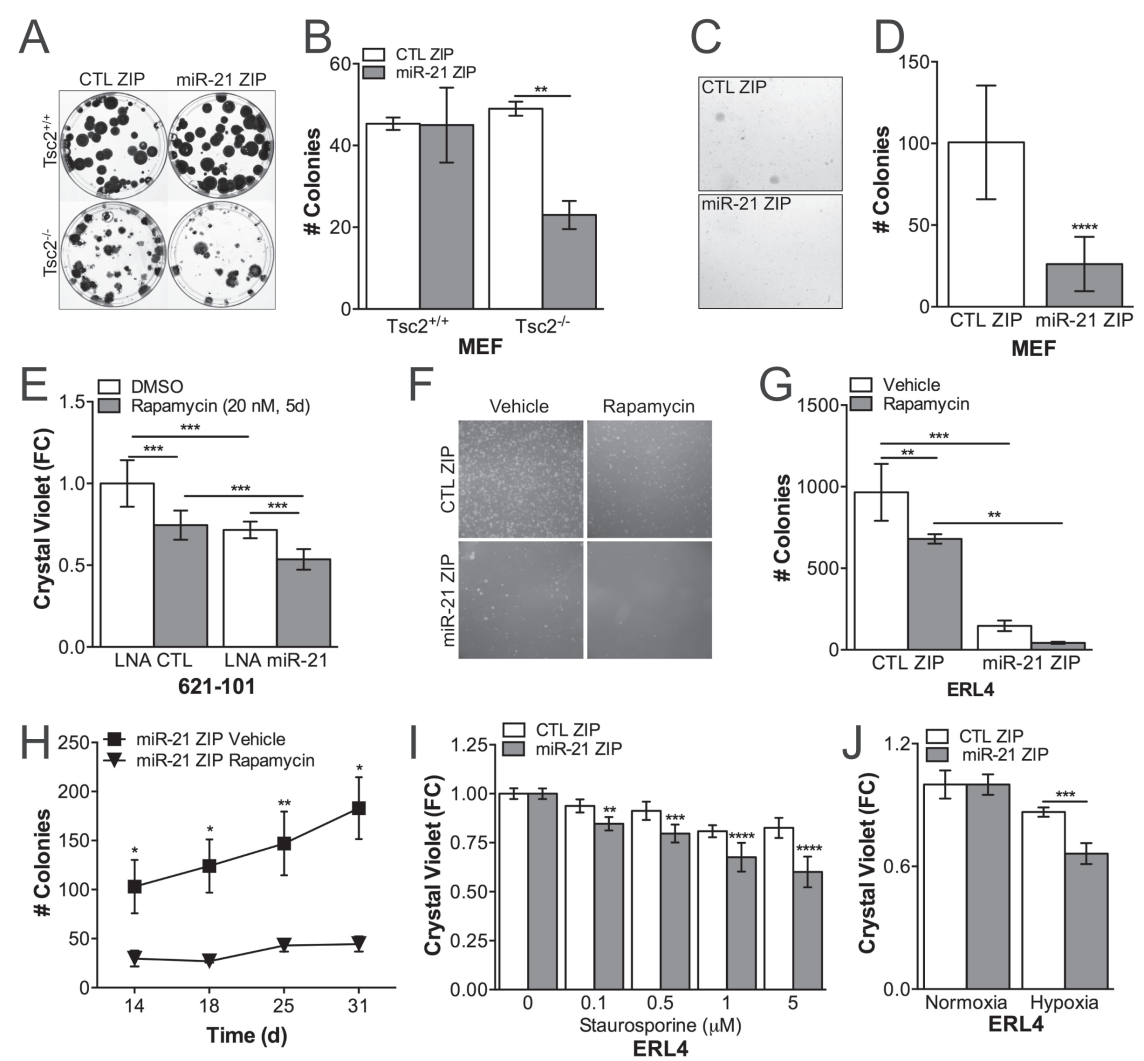
miR-21 promotes proliferation and apoptosis resistance in Tsc2^-/-^ cells **A.**, Tsc2^+/+^ and Tsc2^-/-^ MEFs stably expressing miR-21 inhibitor or control were seeded 200 cells / 10 cm dish and stained with crystal violet at 4 weeks. **B.**, miR-21 inhibition prevented clonogenic growth in Tsc2^-/-^ MEFs from A. **C.**, Tsc2^-/-^ MEFs stably expressing miR-21 inhibitor or control were grown in soft agar for 4 weeks. **D.**, miR-21 inhbition suppressed soft agar growth from C. **E.**, Transient inhibition of miR-21 by locked nucleic acid (LNA) antagomiR against miR-21 suppressed growth of human angiomyolipoma derived 621-101 cells with an additive growth suppression by rapamycin (20nM, 5d) compared to nontargeting control. **F.**, Representative images of colonies from d17 of Tsc2-deficient rat uterine leiomyoma cells stably expressing miR-21 inhibitor, which were grown in soft agar and treated with rapamcyin (10 nM) for 1 week (d 1-7) and then allowed to grow without rapamycin for an additional 3-4 weeks (d 8-31). **G.**, miR-21 inhibition signficantly suppresses growth in soft agar and increases the durability of the rapamycin response 18 days after rapamycin cessation (d25). **H.**, Growth kinetics of ERL4 cells stably expressing miR-21 inhibitor in soft agar after rapamycin cessation compared to vehicle controls. I and J, miR-21 inhibition sensitized cells to staurosporine (6h) and hypoxia (24h). Data are mean +/- SD. Statistical signficance was determined by One-Way ANOVA and Two-Way ANOVA with Bonferroni correction and *p < 0.05, **p < 0.01, ***p < 0.001, ****p < 0.0001.

Tsc2^+/+^ and Tsc2^-/-^ MEFs stably expressing a miR-21 antagomiR (miR-21 ZIP) or control (CTL ZIP) were seeded to assess clonogenic capacity (200 cells into a 10cm dish). At the end of 4 weeks, the colonies were stained with crystal violet and quantified. miR-21 ZIP repressed clonogenic capacity by ∼50% (*p* < 0.01) in a Tsc2-dependent manner, affecting the clonogenic capacity of the Tsc2^-/-^ but not the Tsc2^+/+^ MEFs (Figure 2A, 2B). miR-21 ZIP also repressed anchorage-independent growth in soft agar assays of Tsc2^-/-^ MEFs by 75% (*p* < 0.0001) compared to CTL ZIP cells (Figure 2C, 2D). Since miR-21 is significantly induced by rapamycin, we hypothesized that miR-21 inhibition would synergize with rapamycin treatment in TSC2-deficient 621-101 cells. In proliferation assays, rapamycin alone or miR-21 inhibition with a transient locked nucleic acid (LNA) antagamiR alone reduced proliferation by ∼20% (*p* < 0.001), while combined treatment reduced proliferation by ∼40% over 5 days (*p* < 0.001; Figure 2E). These data suggest an additive effect of combined miR-21 and rapamycin treatment in Tsc2-deficient cells *in vitro*.

We next assessed the effects of miR-21 inhibition with rapamycin treatment followed by rapamycin cessation in soft agar assays using the ERL4 Tsc2-deficient cell line. Cells were seeded in soft agar and treated with rapamycin (10 nM) for the first week, the media was removed and the colonies formed in the absence of rapamycin for the next 3 weeks. While rapamycin repressed colony numbers by ∼30% (*p* < 0.01), miR-21 inhibition reduced the growth of the cells by > 75% (*p* < 0.001) and treatment of the miR-21 ZIP cells with rapamycin for one week at the start of growth in soft agar repressed colony formation by ∼90% (*p* < 0.0001; Figure 2F, 2G). Combined miR-21 inhibition and transient rapamycin treatment inhibited colony formation and growth, as rapamycin significantly reduced the rate of colony growth in the ERL4 cells expressing miR-21 ZIP over the final two weeks of growth in soft agar (Figure 2H). Finally, we found that miR-21 inhibition sensitized Tsc2-deficient cells to the apoptosis inducing agent staurosporine (6h; Figure 2I) and to hypoxia (1% O_2_, 24h, Figure 2J). In aggregate, these data indicate that miR-21 is critical to the proliferation, survival and transformation characteristics of Tsc2-deficient cells. Furthermore, miR-21 inhibition enhances the durability of the response to rapamycin.

### Network-based RNAseq analysis implicates miR-21 in mitochondrial processes

**Figure 3:**
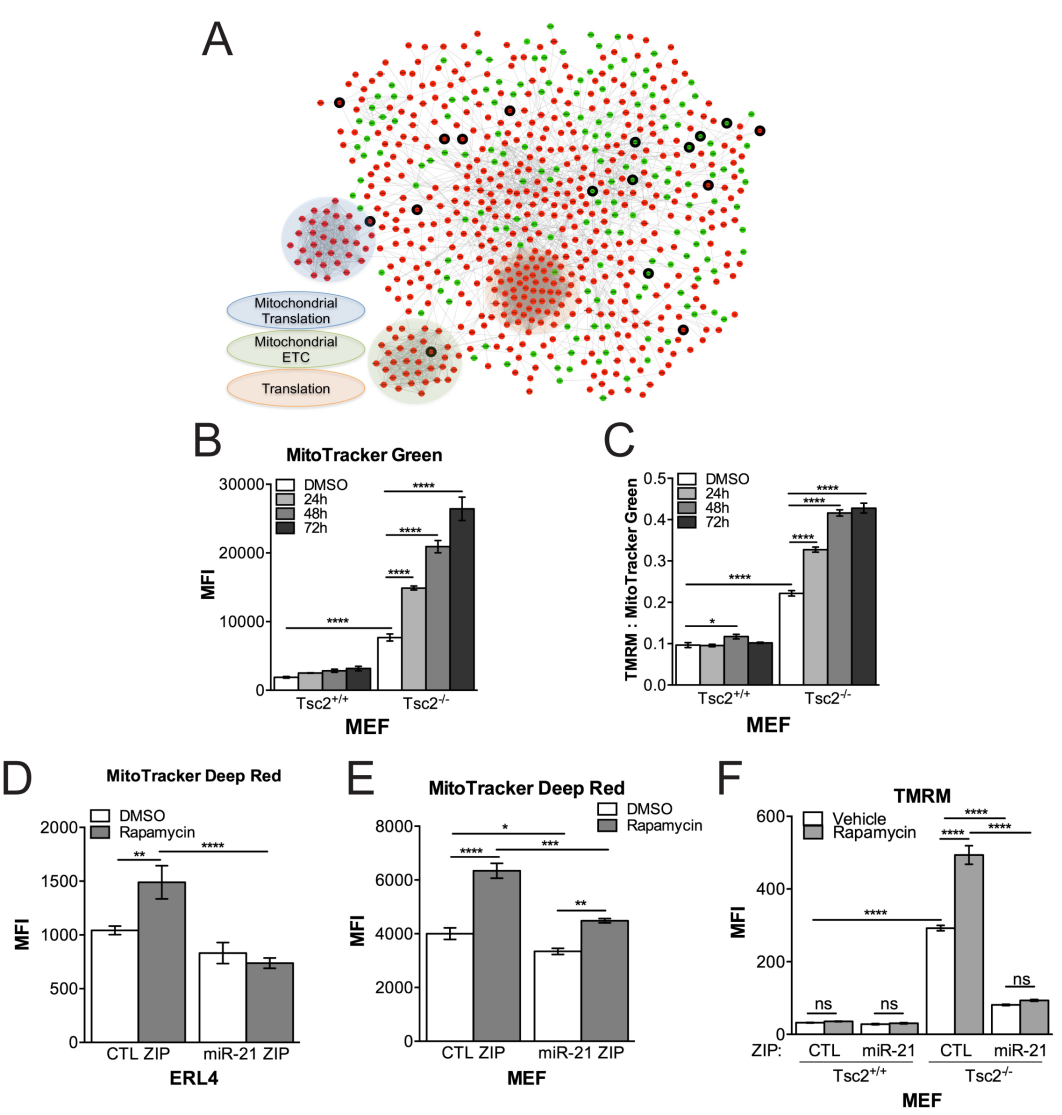
Network-based RNAseq analysis implicates miR-21 in mitochondrial processes **A.**, Network analysis using the consolidated interactome of differentially expressed genes between miR-21 ZIP and CTL ZIP MEFs treated with rapamcyin (20nM, 24h). red upregulated, green-downregulated, thick border- predicted targets of miR-21. **B.**, Mitochondrial content was increased in Tsc2^-/-^ MEFs treated with rapamycin (20 nM) in a time dependent manner in contrast to Tsc2^+/+^ MEFs. **C.**, Rapamycin increases mitochondrial polarization in Tsc2^-/-^ MEFs as assessed by TMRM normalized to MitoTracker Green. **D.**, **E.**, Cells stably expressing a miR-21 inhibitor have lower mitochondria content by MitoTracker Deep Red particularly following rapamycin in ERL4 and MEF cells.F, miR-21 inhibition reduces mitochondrial membrane polarization. Data presented as mean+/- standard deviation. Statistical significance was assessed by Two-Way and One-Way ANOVAs with Bonferroni correction with *p < 0.05, **p < 0.01, ***p < 0.001, ****p < 0.0001.

In order to identify direct targets of miR-21, we performed transcriptional profiling by RNAseq in miR-21 ZIP and CTL ZIP MEFs treated with rapamycin, followed by pathway enrichment analysis on the differentially expressed genes between miR-21 ZIP and CTL ZIP (FC > 1.25, *p* < 0.05; Table 1). Expression profiling highlights the role of miR-21 in various processes involving mRNA, nucleotides, mitochondrial apoptotic signaling, cellular respiration and oxidative stress. To further discern and visualize the enrichment of miR-21-dependent genes and their network connections, we mapped the functional connections among these differentially expressed genes (Supplementary Table S1), using a consolidated set of databases cataloguing known functional interactions in human cells (the “consolidated interactome”, CI). *Via* a spectral partitioning analysis to define densely connected modules within this miR-21-specific gene network, these data showed an unexpected upregulation of genes involved in ribosomes, mitochondria electron transport chain and mitochondrial ribosomes upon miR-21 inhibition (Figure 3A).

**Table 1:** Pathway enrichment analysis of genes differentially expressed following miR-21 inhibition in rapamycin treated Tsc2-deficient cells.

Description	GeneRatio	BgRatio	*p*value	p.adjust	*q*value
ribonucleoprotein complex biogenesis	138/4009	342/20949	5.21E-20	2.73E-16	2.12E-16
ribosome biogenesis	101/4009	224/20949	5.35E-19	1.40E-15	1.09E-15
ncRNA metabolic process	132/4009	372/20949	5.82E-14	1.02E-10	7.90E-11
rRNA processing	67/4009	147/20949	2.32E-13	3.04E-10	2.36E-10
RNA splicing	115/4009	316/20949	3.50E-13	3.68E-10	2.85E-10
ncRNA processing	103/4009	273/20949	4.53E-13	3.97E-10	3.08E-10
mRNA metabolic process	157/4009	482/20949	9.30E-13	6.98E-10	5.42E-10
regulation of mitotic cell cycle	145/4009	436/20949	1.18E-12	7.76E-10	6.03E-10
rRNA metabolic process	67/4009	152/20949	1.60E-12	9.34E-10	7.25E-10
negative regulation of intracellular signal transduction	144/4009	441/20949	6.66E-12	3.49E-09	2.71E-09
cell cycle phase transition	109/4009	313/20949	3.35E-11	1.48E-08	1.15E-08
regulation of protein stability	80/4009	206/20949	3.39E-11	1.48E-08	1.15E-08
ubiquitin-dependent protein catabolic process	155/4009	496/20949	4.68E-11	1.89E-08	1.47E-08
regulation of apoptotic signaling pathway	124/4009	373/20949	5.21E-11	1.95E-08	1.52E-08
mRNA processing	129/4009	395/20949	8.19E-11	2.87E-08	2.23E-08
intrinsic apoptotic signaling pathway	95/4009	274/20949	7.84E-10	2.57E-07	2.00E-07
mitotic cell cycle phase transition	100/4009	294/20949	9.35E-10	2.74E-07	2.13E-07
response to oxidative stress	109/4009	329/20949	9.39E-10	2.74E-07	2.13E-07
regulation of cell cycle phase transition	81/4009	224/20949	1.47E-09	4.07E-07	3.16E-07
cellular respiration	54/4009	130/20949	3.12E-09	7.89E-07	6.13E-07
nucleoside triphosphate metabolic process	78/4009	216/20949	3.16E-09	7.89E-07	6.13E-07
protein import	103/4009	312/20949	3.35E-09	7.99E-07	6.21E-07
ribosomal small subunit biogenesis	29/4009	52/20949	4.59E-09	9.91E-07	7.70E-07
positive regulation of transferase activity	146/4009	488/20949	4.63E-09	9.91E-07	7.70E-07
glycosyl compound metabolic process	100/4009	302/20949	4.72E-09	9.91E-07	7.70E-07
purine nucleoside triphosphate metabolic process	73/4009	200/20949	5.88E-09	1.18E-06	9.16E-07
nucleoside monophosphate metabolic process	77/4009	215/20949	6.06E-09	1.18E-06	9.16E-07
regulation of mitotic cell cycle phase transition	75/4009	208/20949	6.77E-09	1.27E-06	9.86E-07
nucleoside metabolic process	96/4009	289/20949	7.95E-09	1.44E-06	1.12E-06
peptidyl-lysine modification	99/4009	301/20949	8.32E-09	1.45E-06	1.13E-06
ossification	118/4009	377/20949	8.57E-09	1.45E-06	1.13E-06
RNA splicing	70/4009	191/20949	9.86E-09	1.57E-06	1.22E-06
mRNA splicing	70/4009	191/20949	9.86E-09	1.57E-06	1.22E-06
cell cycle checkpoint	63/4009	166/20949	1.14E-08	1.76E-06	1.37E-06
purine nucleoside metabolic process	88/4009	260/20949	1.22E-08	1.79E-06	1.39E-06
ribonucleoprotein complex assembly	64/4009	170/20949	1.26E-08	1.79E-06	1.39E-06
RNA splicing	70/4009	192/20949	1.26E-08	1.79E-06	1.39E-06
negative regulation of cell cycle	113/4009	360/20949	1.48E-08	2.04E-06	1.59E-06
ribonucleoside monophosphate metabolic process	73/4009	204/20949	1.55E-08	2.08E-06	1.62E-06
purine ribonucleoside monophosphate metabolic process	72/4009	201/20949	1.84E-08	2.42E-06	1.88E-06

Since many of the transcriptomic data implicated miR-21 in mitochondrial processes, we next investigated the effects of Tsc2, rapamycin and miR-21 on mitochondrial function. Consistent with prior literature [20, 21], Tsc2-deficient cells had a greater mitochondrial content as assessed by flow cytometry for MitoTracker Green (Figure 3B). Unexpectedly, not only did the mitochondrial content further increase following rapamycin treatment of Tsc2-deficient cells (Figure 3B), but rapamycin increased overall mitochondrial polarization as assessed by TMRM staining (Figure 3C).

We next proceeded to determine if any of the mitochondrial phenotypes observed in the Tsc2-deficient cells were miR-21 dependent. miR-21 inhibition modestly reduced overall mitochondrial content and suppressed the increase in mitochondria observed with rapamycin treatment in ERL4 and MEF cells (Figure 3D, 3E). Inhibition of miR-21 also reduced mitochondrial membrane polarization in the Tsc2-deficient MEF cells (Figure 3F).

In aggregate, miR-21 appears to contribute to the survival and proliferation of Tsc2-deficient cells through regulation of transcripts involved in mitochondrial processes. miR-21 may promote Tsc2-deficient cell survival by maintaining mitochondrial polarization and adaptation to rapamycin in particular.

### miR-21 regulates mitochondrial function in Tsc2-deficient cells

**Figure 4:**
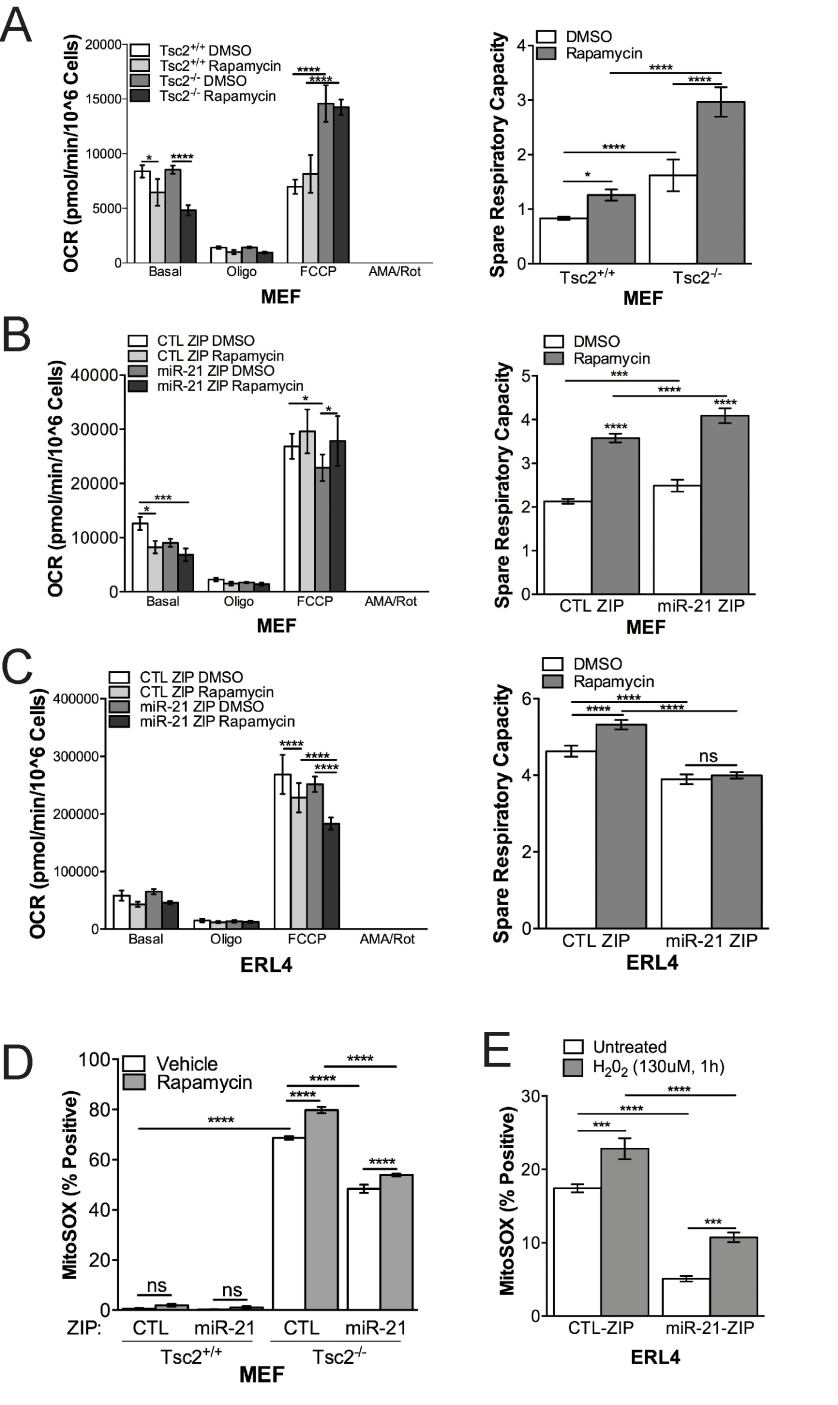
miR-21 inhibition reduces Tsc2^-/-^ cell mitochondrial function **A.**,Tsc2^+/+^ and Tsc2^-/-^ MEFs treated with rapamcyin (20 nM, 24h) or vehicle have distinct mitochondria function by Seahorse XF Analyzer MitoStress Test Assay. Tsc2^-/-^ MEFs have higher maximal respiration capacity, which is not reduced by rapamycin (20 nM, 24h). Right panel, Spare respiratory capacity is higher in Tsc2^-/-^ MEFs and increased by rapamycin in contrast to Tsc2^+/+^ MEFs. **B.** & **C.** (left panels), Stable miR-21 inhibition reduces mitochondrial function under basal or FCCP conditions in MEFs or ERL4 cells. B & C (right panels), miR-21 inhibition reduces spare respiratory capacity in the MEFs and ERL4 cells. **D.** & **E.**, miR-21 inhibition reduces the production of mitochondrial ROS. Data presented as mean+/- standard deviation. Statistical significance was assessed by Two-Way and One-Way ANOVAs with Bonferroni correction with *p < 0.05, **p < 0.01, ***p < 0.001, ****p < 0.0001.

Since miR-21 inhibition reduced mitochondrial content and polarization, particularly in response to rapamycin, we next investigated mitochondrial function in Tsc2-deficient and wildtype cells using the Seahorse XF Analyzer Mitostress Test Assay. Rapamycin repressed oxygen consumption rate basally, while the Tsc2-deficient cells had an increased maximal respiration rate following FCCP treatment (Figure 4A, left panel). Consistent with the TMRM and MitoTracker data, rapamycin increased spare respiratory capacity particularly in the Tsc2-deficient cells (Figure 4A, right panel).

miR-21 inhibition in combination with rapamycin repressed basal OCR in MEFs (Figure 4B, left panel) and maximal respiration in ERL4 cells (Figure 4C, left panel). The impact of miR-21 on basal and maximal respiration impacted the spare respiratory capacity of the cells, which was unexpectedly increased by miR-21 inhibition in the MEFs, but decreased by miR-21 inhibition in the ERL4 cells (Figure 4B, 4C, right panels, respectively). Finally, MitoSOX positive cells were also significantly reduced in miR-21 ZIP in MEFs (∼25%, *p* < 0.0001) and ERL4 cells (∼70%, *p* < 0.0001) (Figure 4D, 4E).

These data suggest that miR-21 has a significant impact on mitochondrial function and the cellular response to rapamycin at the level of mitochondrial function. Overall, these data further support the concept that miR-21 is a critical regulator of mitochondrial homeostasis in Tsc2-deficient cells.

### miR-21 promotes Tsc2-deficient tumor growth *in vivo*

**Figure 5:**
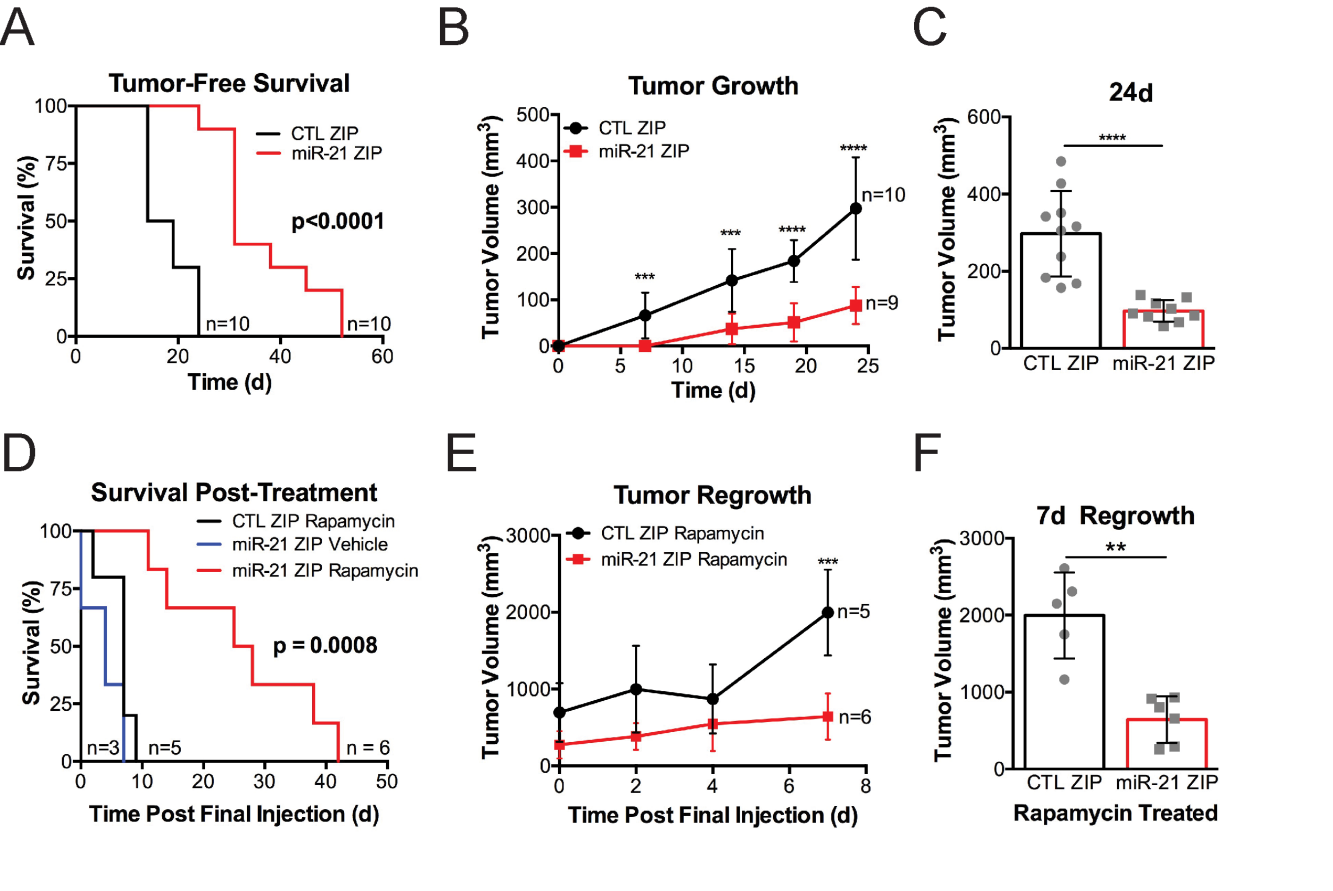
miR-21 promotes the growth of Tsc2^-/-^ ERL4 xenografts **A-F**, Mice were subcutaneously injected with 2X10^6 ERL4 cells expressing miR-21 ZIP or CTL ZIP. **A.**,Tumor bearing mice were identified when tumors were hard upon palpation and greater than 100 mm3. **B.**, All mice that achieved tumor bearing status within 24 days of cell injection were included in tumor volume analysis. **C.**, Scatter plot showing individual tumors at day 24 post cell injection. **D.**-**F.**, CTL-ZIP and miR-21-ZIP tumors 300-400 mm^3^ in size were assigned to vehicle or rapamycin treatment (3mg/kg, 3 days/week for 4 weeks). **D.**, Survival was assessed after rapamycin cessation. Mice were removed from the group when tumors achieved a volume of 1500mm3. None of the vehicle treated CTL-ZIP mice survived the treatment phase. **E.**, Tumor volume in CTL-ZIP and miR-21 ZIP was assessed in the 7 days following rapamycin cessation. **F.**, Scatter plot of individual tumors 7 days after rapamycin cessation in the CTL-ZIP and miR-21 ZIP mice. Data presented as mean+/- standard deviation. Statistical significance was assessed in survival analysis by Mantel-Cox Test or Students Unpaired T test for remaining data sets with **p < 0.01, ***p < 0.001, ****p < 0.0001.

To determine the dependence of Tsc2-deficient cells on miR-21 *in vivo,* we generated ERL4 xenografts expressing either miR-21 ZIP or CTL ZIP in mice. miR-21 ZIP significantly increased tumor-free survival, with CTL ZIP mice developing tumors by 25 days while miR-21 ZIP tumors became palpable up to 50 days (*p* < 0.0001; Figure 5A). Inhibition of miR-21 significantly repressed the rate of tumor growth (Figure 5B) and the miR-21 ZIP tumors were approximately half the size of the CTL ZIP tumors at 24 days (Figure 5C).

To assess combined rapamycin treatment with miR-21 inhibition *in vivo*, we treated tumors once they were 300-400 mm^3^ with rapamycin (3mg/kg, MWF) for 4 weeks. We then assessed survival off treatment as tumors achieved the > 1500 mm^3^ endpoint. CTL ZIP rapamycin treated mice and miR-21 ZIP vehicle treated mice survived approximately 7-10 days after treatment cessation, while mice bearing miR-21 ZIP xenografts treated with rapamycin survived up to 40 days after treatment cessation (*p* = 0.008; Figure 5D). Tumor growth rate was significantly slower in the miR-21 ZIP xenografts compared to the CTL ZIP xenografts following rapamycin cessation (Figure 5E). Following 7 days of regrowth, the miR-21 ZIP tumors were ∼50% smaller than the CTL ZIP tumors (*p* < 0.01; Figure 5F).

These data suggest that miR-21 supports the tumorigenic growth of Tsc2-deficient cells *in vivo* and provide evidence that combined miR-21 inhibition with rapamycin treatment may increase the efficacy of mTORC1 inhibition.

## DISCUSSION

The role of microRNA in the pathogenesis and therapy of TSC/LAM remains largely unexplored. We have found that miR-21, an oncogenic miRNA, is increased 10-fold in Tsc2-deficient cells compared to Tsc2^+/+^ control cells (Figure 1A). Prior studies have shown that miR-21 is increased in brain lesions of TSC patients [19], and that miR-21 is a “Rapa-miR” which is regulated by mTORC1 [13]. Despite these findings, the dependence of mTORC1-driven tumors on miR-21 has not been previously investigated. We report here that miR-21 is a critical mediator of tumorigenesis in Tsc2-deficient cells. These data provide the first evidence that miR-21 is a candidate therapeutic target for tumors in TSC patients, with the potential for particularly high efficacy when combined with an mTORC1 inhibitor.

We found that downregulation of miR-21 in TSC2-deficient cells decreases colony formation and increases sensitivity to staurosporine-induced apoptosis and hypoxia. *In vivo*, inhibition of miR-21 delayed tumors and decreased tumor volume by > 50% (*p* < 0.0001). Importantly, miR-21 inhibition also strikingly repressed tumor regrowth following release from rapamycin therapy, with the median survival of mice after rapamycin discontinuation rising from one week in the control group to four weeks in the miR-21 antagonist group. This has relevance to the therapy of tumors in TSC patients, in whom rapalogs have efficacy in partially decreasing tumor size, but the tumors regrow upon treatment discontinuation. Therapeutic strategies that enhance the response to rapalogs and enable treatment-free intervals could benefit TSC patients, especially young children, who currently receive life-long rapalog therapy.

To elucidate more completely the mechanisms through which miR-21 exerts its effects, we used expression profiling in combination with network analysis, as performed in prior studies of miR-21 and other miRNAs in distinct contexts [22, 23]. Such network analysis revealed nodes related to translation of both mitochondrial and nuclear genes and the mitochondrial electron transport chain.

Gene set enrichment analysis comparing Tsc2-deficient cells expressing miR-21 inhibitor to control in the context of rapamycin revealed that miR-21 has a significant impact on mRNA and translation associated processes. Both cytoplasmic and mitochondrial ribosomal transcripts are upregulated, which is intriguing considering that rRNA is a critical sink for nucleotides [24, 25]. We also observed upregulation of components of the electron transport chain and intrinsic apoptosis pathways. Importantly, we found that rapamycin increased mitochondrial content and polarization, and these effects of rapamycin were miR-21 dependent. Prior studies have shown that TSC2-deficient cells have increased mitochondrial number, which is believed to be the consequence of impaired mitophagy, and rapamycin has been shown to increase mitochondrial fitness in neuronal models of TSC [20, 21, 26]. These data suggest that miR-21 maintains mitochondrial homeostasis, a critical determinant of tumorigenic potential in mTORC1 hyperactivated cells [27]. Interestingly, miR-21 inhibition has been shown to protect mitochondria and limit oxidative stress in renal fibrosis models [28-30].

We were surprised to find that miR-21 is higher in TSC2-deficient cells, and yet is further increased by rapamycin both *in vitro* and *in vivo*. We infer, therefore, that miR-21 is not regulated by TSC2 *via* the canonical mTORC1-dependent pathways. A variety of non-canonical functions of TSC2 and Rheb have been reported, although these remain incompletely understood [6]. miR-21 was previously shown to be a target of both STAT3 [31] and AKT [32, 33], both of which are impacted by mTORC1 activity, but these are presumably not the mechanisms through which miR-21 is regulated since these pathways would be predicted to be mTORC1 dependent. miR-21 is located within an intron of the TMEM49 protein, which has been implicated in autophagy, a process activated by rapamycin, but again this type of regulatory mechanism would be predicted to be mTORC1-dependent. Further studies will be required to elucidate the precise molecular mechanism underlying miR-21 upregulation in TSC2-deficient cells.

Clinical trials are currently underway targeting miR-21 with bioavailable antagomiRs. Our data suggest that tumors in TSC and LAM patients, as well as sporadic cancers associated with mTORC1 hyperactivation, may benefit from miR-21 targeted strategies, particularly when combined with rapalog therapy.

## MATERIALS AND METHODS

### Cell lines and culture

Tsc2^-/-^p53^-/-^ and Tsc2^+/+^p53^-/-^ mouse embryonic fibroblasts (MEFs) were authenticated and provided by David Kwiatkowski (Brigham and Women’s Hospital, Boston, MA). The MEFs were obtained in 2005 and stocks were frozen at passage 8. Tsc2-deficient ELT3 cells, which were isolated from an Eker rat uterine leiomyoma, were provided by Cheryl Walker [34]. We introduced a luciferase cell reporter to produce the ERL4 cell line [35]. The miR-21 ZIP or CTL ZIP were introduced by lentivirus into the MEFs or the ERL4 cells and experiments were performed between passages 15-30. Cells were FACS sorted for GFP expression by the ZIP construct and maintained in puromycin (5 μg/mL). ZIP expression was validated repeatedly by flow cytometry. The 621 human-derived angiomyolipoma cells were isolated in 2004 by our group [36]. We obtained aliquots of immortalized 621 (621-101) cells from The Rothberg Institute and used the cells at passage 10-30 with genetic validation of the TSC2 mutation. Cell lines were validated by immunoblot analysis for Tsc2 expression and phosphorylation of the ribosomal subunit S6 (235/236) in serum free conditions. MycoAlert (Lonza) was used to detect mycoplasma contamination and cells were re-tested monthly. Cells were cultured at 37°C in 5% CO_2_ in DMEM containing 4.5g/L glucose, pyruvate, and glutamine supplemented with 10% FBS and gentamycin sulfate (50 μg/mL).

### Chemicals and miR-21 antagomiRs

Rapamycin was purchased from LC Laboratories. Transient miR-21 inhibition was achieved using locked nucleic acids (LNA) from Exiqon in comparison to a non-targeting control oligonucleotide. Stable miR-21 inhibition was achieved using a lentivirus construct with a GFP reporter expressing either miR-21 ZIP or CTL ZIP from System Biosciences, Inc.

### Crystal violet assay

Cells were seeded at 1,000 cells/well in 96-well plates and processed for crystal violet at the indicated time points. Cells were fixed for 5 minutes with 10% formalin and then stained with 0.5% crystal violet in distilled water for 20 minutes. Crystal violet was removed and cells were washed with H_2_O. Absorbance was read at 540 nm after the plates dried completely and crystal violet was dissolved using 100 uL of methanol.

### Soft Agar colony formation assay

MEFs (75,000 cells/well) were suspended in 0.4% Difco Noble Agar (BD 214220) diluted in DMEM and seeded (2 ml/well) over a 0.6% agar diluted in DMEM bottom layer previously solidified on 6-well plates. Colonies were quantified from images taken during the 4 weeks of culture with a dissecting microscope using the Colony Counter ImageJ macro (Size 100-30,000; Circularity 0.5-1).

### MitoStress test

Oxygen consumption rate was measured using the Seahorse Bioscience XF^e^24 analyzer using the standard MitoStress Test kit, Oligomycin (1uM) FCCP (1.5uM) Antimycin/Rotenone (0.5uM). Oxygen consumption rate was normalized to cell number.

### Mitochondrial Staining and flow cytometry

Cells were incubated in 5 uM MitoTracker Green (Molecular Probes, Thermo Fisher) and 20 nM Tetramethylrhodamine, Methyl Ester (TMRM) or 5 uM MitoTracker Deep Red FM (Molecular Probes, Thermo Fisher) in serum-free, phenol red-free DMEM for 15-20 minutes. The cells were then trypsinized and recovered in 2% serum phenol red-free DMEM and centrifuged 425 xg for 2 minutes. The pelleted cells were resuspended in 300 ul of serum-free, phenol red-free DMEM and kept on ice. Fluorescence was assessed by flow cytometry (BD FACS Canto II, BD Biosciences), and analyzed with FlowJo analytical software (Treestar).

### Gene-set enrichment analysis

To identify pathways associated with miR-21 inhibition (Table 1), gene set analysis using the Generally Applicable Gene-set Enrichment for Pathway Analysis (GAGE) tool (v2.22.0) was performed. The GAGE analysis used the log2 fold changes output from DESeq2 to identify significantly dysregulated KEGG pathways (*q*-value < 0.05). Following GAGE analysis, the Pathview tool (v1.12.0) was used to map the pathway data from GAGE onto KEGG pathway graphs for pathway visualizations.

### Network analysis

To generate the miR-21 network, direct functional connections among genes differentially expressed between miR-21 ZIP *vs* CTL ZIP in Tsc2^-/-^ MEFs treated with rapamycin (FC > 1.5, *p* < 0.05) were mapped using compiled data from a catalog (a “consolidated interactome” or CI) of known functional human gene and protein interactions. This CI combined data from BioGRID [37], CORUM [38], DIP [39], InnateDB [40], IntAct [41], MatrixDB [42], and MINT [41, 43], and in total contains 12,469, genes and 113,689 interactions. Importantly, interactions represented a wide range of functional relationships including transcriptional, translational, and protein level associations. The resulting miR-21 network contained 1,210 genes and 3,595 interactions, with 699 genes and 3,557 interactions forming a distinct largest connected component (LCC). In order to more clearly visualize the role of miR-21 in regulating this cohort of genes, we identified direct miR-21 targets as reported by one of three miRNA target prediction algorithms (TargetScan, PicTar, and Diana microT) and mapped these predictions onto the network.

### Animal studies

All animal studies were performed in accordance with institutional protocols approved by Boston Children’s Hospital Animal Care and Use Committee. Xenografts were generated by injecting (2.0-2.5x10^6) cells mixed cell suspension to matrigel 1:1 (v/v) to a final volume of 100-150ul bilaterally or unilaterally into the shoulders of anesthetized, immunocompromised mice using a 21G needle. The 621-101 tumors were generated in triple immunodeficient NOD.Cg-Prkdc ^scid^IL2rg^m12ji^ /SzJ (NSG) mice obtained from Jackson Laboratories. ERL4 tumors were generated by injecting cells into Nu/Nu nude mice obtained from Charles River. Mice were inspected weekly and tumors were measured once a week by caliper once they became palpable and > 100mm^3^. Mice were treated with rapamycin once tumors reached 300-400mm^3^, at 3 mg/kg MWF for 1-4 weeks. Tumors were then monitored three times a week.

### Statistical analyses

*In vivo* data are presented as the mean +/- 95% confidence interval (CI) and *in vitro* studies are presented as the mean +/- standard deviation (SD). Normally distributed data were analyzed for statistical significance with Student’s unpaired *t*-test and multiple comparisons were made with one-way and two-way ANOVAs with Bonferroni correction. Alternatively, nonparametric Kruskal-Wallis and Dunn’s correction were used (GraphPad Prism version 6 for Mac; GraphPad Software, www.graphpad.com). Statistical significance was defined as *p* < 0.05.

## SUPPLEMENTARY MATERIALS TABLE



## Abbreviations

TSC: Tuberous sclerosis complex
LAM: lymphangioleiomyomatosis
mTORC1: mammalian target of rapamycin complex 1
miRNA or miRs: microRNAs
TCGA: The Cancer Genome Atlas
MEF: mouse embryonic fibroblasts
OCR: oxygen consumption rate

## References

[1] Crino PB, Nathanson KL, Henske EP (2006). The tuberous sclerosis complex. N Engl J Med.

[2] Henske EP, Jóźwiak S, Kingswood JC, Sampson JR, Thiele EA (2016). Tuberous sclerosis complex. Nat Rev Dis Primers.

[3] Carsillo T, Astrinidis A, Henske EP (2000). Mutations in the tuberous sclerosis complex gene TSC2 are a cause of sporadic pulmonary lymphangioleiomyomatosis. Proc Natl Acad Sci USA.

[4] Saxton RA, Sabatini DM (2017). mTOR Signaling in Growth, Metabolism, and Disease. Cell.

[5] Gomes AP, Blenis J (2015). A nexus for cellular homeostasis: the interplay between metabolic and signal transduction pathways. Curr Opin Biotechnol.

[6] Neuman NA, Henske EP (2011). Non-canonical functions of the tuberous sclerosis complex-Rheb signalling axis. EMBO Mol Med.

[7] Bissler JJ, McCormack FX, Young LR, Elwing JM, Chuck G, Leonard JM, Schmithorst VJ, Laor T, Brody AS, Bean J, Salisbury S, Franz DN (2008). Sirolimus for angiomyolipoma in tuberous sclerosis complex or lymphangioleiomyomatosis. N Engl J Med.

[8] Bissler JJ, Kingswood JC, Radzikowska E, Zonnenberg BA, Frost M, Belousova E, Sauter M, Nonomura N, Brakemeier S, de Vries PJ, Whittemore VH, Chen D, Sahmoud T (2013). Everolimus for angiomyolipoma associated with tuberous sclerosis complex or sporadic lymphangioleiomyomatosis (EXIST-2): a multicentre, randomised, double-blind, placebo-controlled trial. Lancet.

[9] Franz DN, Belousova E, Sparagana S, Bebin EM, Frost M, Kuperman R, Witt O, Kohrman MH, Flamini JR, Wu JY, Curatolo P, de Vries PJ, Whittemore VH (2013). Efficacy and safety of everolimus for subependymal giant cell astrocytomas associated with tuberous sclerosis complex (EXIST-1): a multicentre, randomised, placebo-controlled phase 3 trial. Lancet.

[10] Krueger DA, Care MM, Holland K, Agricola K, Tudor C, Mangeshkar P, Wilson KA, Byars A, Sahmoud T, Franz DN (2010). Everolimus for subependymal giant-cell astrocytomas in tuberous sclerosis. N Engl J Med.

[11] Young L, Lee HS, Inoue Y, Moss J, Singer LG, Strange C, Nakata K, Barker AF, Chapman JT, Brantly ML, Stocks JM, Brown KK, Lynch JP (2013). Serum VEGF-D a concentration as a biomarker of lymphangioleiomyomatosis severity and treatment response: a prospective analysis of the Multicenter International Lymphangioleiomyomatosis Efficacy of Sirolimus (MILES) trial. Lancet Respir Med.

[12] Chen CZ (2005). MicroRNAs as oncogenes and tumor suppressors. N Engl J Med.

[13] Trindade AJ, Medvetz DA, Neuman NA, Myachina F, Yu J, Priolo C, Henske EP (2013). MicroRNA-21 is induced by rapamycin in a model of tuberous sclerosis (TSC) and lymphangioleiomyomatosis (LAM). PLoS One.

[14] Li X, Huang K, Yu J (2014). Inhibition of microRNA-21 upregulates the expression of programmed cell death 4 and phosphatase tensin homologue in the A431 squamous cell carcinoma cell line. Oncol Lett.

[15] Talotta F, Cimmino A, Matarazzo MR, Casalino L, De Vita G, D’Esposito M, Di Lauro R, Verde P (2009). An autoregulatory loop mediated by miR-21 and PDCD4 controls the AP-1 activity in RAS transformation. Oncogene.

[16] Zhang H, Guo Y, Shang C, Song Y, Wu B (2012). miR-21 downregulated TCF21 to inhibit KISS1 in renal cancer. Urology.

[17] Medina PP, Nolde M, Slack FJ (2010). OncomiR addiction in an in vivo model of microRNA-21-induced pre-B-cell lymphoma. Nature.

[18] Creighton CJ, Morgan M, Gunaratne PH, Wheeler DA, Gibbs RA, Gordon Robertson A, Chu A, Beroukhim R, Cibulskis K, Signoretti S, Vandin Hsin-Ta Wu F, Raphael BJ, Verhaak RG (2013). Comprehensive molecular characterization of clear cell renal cell carcinoma. Nature.

[19] van Scheppingen J, Iyer AM, Prabowo AS, Mühlebner A, Anink JJ, Scholl T, Feucht M, Jansen FE, Spliet WG, Krsek P, Zamecnik J, Buccoliero AM, Giordano F (2016). Expression of microRNAs miR21, miR146a, and miR155 in tuberous sclerosis complex cortical tubers and their regulation in human astrocytes and SEGA-derived cell cultures. Glia.

[20] Goto J, Talos DM, Klein P, Qin W, Chekaluk YI, Anderl S, Malinowska IA, Di Nardo A, Bronson RT, Chan JA, Vinters HV, Kernie SG, Jensen FE (2011). Regulable neural progenitor-specific Tsc1 loss yields giant cells with organellar dysfunction in a model of tuberous sclerosis complex. Proc Natl Acad Sci USA.

[21] Ebrahimi-Fakhari D, Saffari A, Wahlster L, Di Nardo A, Turner D, Lewis TL, Conrad C, Rothberg JM, Lipton JO, Kölker S, Hoffmann GF, Han MJ, Polleux F, Sahin M (2016). Impaired Mitochondrial Dynamics and Mitophagy in Neuronal Models of Tuberous Sclerosis Complex. Cell Reports.

[22] Bertero T, Lu Y, Annis S, Hale A, Bhat B, Saggar R, Saggar R, Wallace WD, Ross DJ, Vargas SO, Graham BB, Kumar R, Black SM (2014). Systems-level regulation of microRNA networks by miR-130/301 promotes pulmonary hypertension. J Clin Invest.

[23] Parikh VN, Jin RC, Rabello S, Gulbahce N, White K, Hale A, Cottrill KA, Shaik RS, Waxman AB, Zhang YY, Maron BA, Hartner JC, Fujiwara Y (2012). MicroRNA-21 integrates pathogenic signaling to control pulmonary hypertension: results of a network bioinformatics approach. Circulation.

[24] Wiegers U, Kramer G, Klapproth K, Hilz H (1976). Separate pyrimidine-nucleotide pools for messenger-RNA and ribosomal-RNA synthesis in HeLa S3 cells. Eur J Biochem.

[25] Donati G, Bertoni S, Brighenti E, Vici M, Treré D, Volarevic S, Montanaro L, Derenzini M (2011). The balance between rRNA and ribosomal protein synthesis up- and downregulates the tumour suppressor p53 in mammalian cells. Oncogene.

[26] Ebrahimi-Fakhari D, Saffari A, Wahlster L, Sahin M (2017). Using tuberous sclerosis complex to understand the impact of MTORC1 signaling on mitochondrial dynamics and mitophagy in neurons. Autophagy.

[27] Dibble CC, Manning BD (2013). Signal integration by mTORC1 coordinates nutrient input with biosynthetic output. Nat Cell Biol.

[28] Chau BN, Xin C, Hartner J, Ren S, Castano AP, Linn G, Li J, Tran PT, Kaimal V, Huang X, Chang AN, Li S, Kalra A (2012). MicroRNA-21 promotes fibrosis of the kidney by silencing metabolic pathways. Sci Transl Med.

[29] Gomez IG, MacKenna DA, Johnson BG, Kaimal V, Roach AM, Ren S, Nakagawa N, Xin C, Newitt R, Pandya S, Xia TH, Liu X, Borza DB (2015). Anti-microRNA-21 oligonucleotides prevent Alport nephropathy progression by stimulating metabolic pathways. J Clin Invest.

[30] Gomez IG, Nakagawa N, Duffield JS (2016). MicroRNAs as novel therapeutic targets to treat kidney injury and fibrosis. Am J Physiol Renal Physiol.

[31] Iliopoulos D, Jaeger SA, Hirsch HA, Bulyk ML, Struhl K (2010). STAT3 activation of miR-21 and miR-181b-1 via PTEN and CYLD are part of the epigenetic switch linking inflammation to cancer. Mol Cell.

[32] Polytarchou C, Iliopoulos D, Hatziapostolou M, Kottakis F, Maroulakou I, Struhl K, Tsichlis PN (2011). Akt2 regulates all Akt isoforms and promotes resistance to hypoxia through induction of miR-21 upon oxygen deprivation. Cancer Res.

[33] Zhang Y, He S, Du X, Jiang Y, Tian B, Xu S (2017). Rapamycin suppresses hypoxia/reoxygenation-induced islet injury by up-regulation of miR-21 via PI3K/Akt signalling pathway. Cell Prolif.

[34] Hodges LC, Houston KD, Hunter DS, Fuchs-Young R, Zhang Z, Wineker RC, Walker CL (2002). Transdominant suppression of estrogen receptor signaling by progesterone receptor ligands in uterine leiomyoma cells. Mol Cell Endocrinol.

[35] Yu JJ, Robb VA, Morrison TA, Ariazi EA, Karbowniczek M, Astrinidis A, Wang C, Hernandez-Cuebas L, Seeholzer LF, Nicolas E, Hensley H, Jordan VC, Walker CL, Henske EP (2009). Estrogen promotes the survival and pulmonary metastasis of tuberin-null cells. Proc Natl Acad Sci USA.

[36] Yu J, Astrinidis A, Howard S, Henske EP (2004). Estradiol and tamoxifen stimulate LAM-associated angiomyolipoma cell growth and activate both genomic and nongenomic signaling pathways. Am J Physiol Lung Cell Mol Physiol.

[37] Chatr-Aryamontri A, Breitkreutz BJ, Oughtred R, Boucher L, Heinicke S, Chen D, Stark C, Breitkreutz A, Kolas N, O’Donnell L, Reguly T, Nixon J, Ramage L (2015). The BioGRID interaction database: 2015 update. Nucleic Acids Res.

[38] Ruepp A, Waegele B, Lechner M, Brauner B, Dunger-Kaltenbach I, Fobo G, Frishman G, Montrone C, Mewes HW (2010). CORUM: the comprehensive resource of mammalian protein complexes—2009. Nucleic Acids Res.

[39] Salwinski L, Miller CS, Smith AJ, Pettit FK, Bowie JU, Eisenberg D (2004). The Database of Interacting Proteins: 2004 update. Nucleic Acids Res.

[40] Breuer K, Foroushani AK, Laird MR, Chen C, Sribnaia A, Lo R, Winsor GL, Hancock RE, Brinkman FS, Lynn DJ (2013). InnateDB: systems biology of innate immunity and beyond—recent updates and continuing curation. Nucleic Acids Res.

[41] Orchard S, Ammari M, Aranda B, Breuza L, Briganti L, Broackes-Carter F, Campbell NH, Chavali G, Chen C, del-Toro N, Duesbury M, Dumousseau M, Galeota E (2014). The MIntAct project—IntAct as a common curation platform for 11 molecular interaction databases. Nucleic Acids Res.

[42] Launay G, Salza R, Multedo D, Thierry-Mieg N, Ricard-Blum S (2015). MatrixDB, the extracellular matrix interaction database: updated content, a new navigator and expanded functionalities. Nucleic Acids Res.

[43] Licata L, Briganti L, Peluso D, Perfetto L, Iannuccelli M, Galeota E, Sacco F, Palma A, Nardozza AP, Santonico E, Castagnoli L, Cesareni G. MINT (2012). the molecular interaction database: 2012 update. Nucleic Acids Res.

